# Anatomically realistic ultrasound phantoms using gel wax with 3D printed moulds

**DOI:** 10.1088/1361-6560/aa9e2c

**Published:** 2018-01-04

**Authors:** Efthymios Maneas, Wenfeng Xia, Daniil I Nikitichev, Batol Daher, Maniragav Manimaran, Rui Yen J Wong, Chia-Wei Chang, Benyamin Rahmani, Claudio Capelli, Silvia Schievano, Gaetano Burriesci, Sebastien Ourselin, Anna L David, Malcolm C Finlay, Simeon J West, Tom Vercauteren, Adrien E Desjardins

**Affiliations:** 1 Department of Medical Physics and Biomedical Engineering, University College London, Gower Street, London WC1E 6BT, United Kingdom; 2 Wellcome/EPSRC Centre for Interventional and Surgical Sciences, University College London, Charles Bell House, 67-73 Riding House Street, London W1W 7EJ, United Kingdom; 3 Translational Imaging Group, Centre for Medical Image Computing, Department of Medical Physics and Biomedical Engineering, University College London, Gower Street, London WC1E 6BT, United Kingdom; 4 Cardiovascular Engineering Laboratory, UCL Mechanical Engineering, University College London, Torrington Place, London WC1E 7JE, United Kingdom; 5 Centre for Cardiovascular Imaging, University College London, Institute of Cardiovascular Science & Cardiorespiratory Unit, Great Ormond Street Hospital for Children, London WC1N 3JH, United Kingdom; 6 Ri.MED Foundation, Bioengineering Group, Palermo, Italy; 7 St Bartholomew’s Hospital, West Smithfield, London EC1 7BE, United Kingdom; 8 Institute for Women’s Health, University College London, 86-96 Chenies Mews, London WC1E 6HX, United Kingdom; 9 Department of Development and Regeneration, KU Leuven (Katholieke Universiteit), Belgium; 10 Department of Anaesthesia, University College Hospital, Main Theatres, Maple Bridge Link Corridor, Podium 3, 235 Euston Road, London NW1 2BU, United Kingdom; 11 These authors contributed equally to this work.; 12 These authors contributed equally to this work.; wenfeng.xia@ucl.ac.uk

**Keywords:** ultrasound phantoms, 3D printing, tissue mimicking materials, interventional procedures

## Abstract

Here we describe methods for creating tissue-mimicking ultrasound phantoms based on patient anatomy using a soft material called gel wax. To recreate acoustically realistic tissue properties, two additives to gel wax were considered: paraffin wax to increase acoustic attenuation, and solid glass spheres to increase backscattering. The frequency dependence of ultrasound attenuation was well described with a power law over the measured range of 3–10 MHz. With the addition of paraffin wax in concentrations of 0 to 8 w/w%, attenuation varied from 0.72 to 2.91 dB cm^−1^ at 3 MHz and from 6.84 to 26.63 dB cm^−1^ at 10 MHz. With solid glass sphere concentrations in the range of 0.025–0.9 w/w%, acoustic backscattering consistent with a wide range of ultrasonic appearances was achieved. Native gel wax maintained its integrity during compressive deformations up to 60%; its Young’s modulus was 17.4  ±  1.4 kPa. The gel wax with additives was shaped by melting and pouring it into 3D printed moulds. Three different phantoms were constructed: a nerve and vessel phantom for peripheral nerve blocks, a heart atrium phantom, and a placental phantom for minimally-invasive fetal interventions. In the first, nerves and vessels were represented as hyperechoic and hypoechoic tubular structures, respectively, in a homogeneous background. The second phantom comprised atria derived from an MRI scan of a patient with an intervening septum and adjoining vena cavae. The third comprised the chorionic surface of a placenta with superficial fetal vessels derived from an image of a post-partum human placenta. Gel wax is a material with widely tuneable ultrasound properties and mechanical characteristics that are well suited for creating patient-specific ultrasound phantoms in several clinical disciplines.

## 1. Introduction

Phantoms are essential clinical training tools for ultrasound imaging, and they are becoming more prominent as the use of this modality for guiding percutaneous procedures increases. Conventional, off-the-shelf commercial phantoms represent a very limited range of tissue structures and they can have anatomical imperfections (Hunt *et al* 2013). Moreover, their high costs preclude routine use as training tools. Several studies have showed promising results from anatomically realistic Doppler flow phantoms (Poepping *et al* 2004, King *et al* 2011). However, obtaining complex patient-specific geometries has been challenging. With the advent of 3D printing, there is a wide range of new methods for creating inexpensive, custom ultrasound phantoms. For instance, these printers can create polymeric structures that represent highly hyperechoic hard tissue structures such as the spine, which can be embedded in a tissue-mimicking material (TMM) (West *et al* 2014). Additionally, they can create specialized moulds for TMMs with which to create anatomically realistic soft tissue structures such as wall-less blood vessels (Nikitichev *et al* 2016), the kidney (Hunt *et al* 2013), and the heart (Holmes *et al* 2008). One of the limiting factors for creating ultrasound imaging phantoms using 3D printing has been a dearth of choices for suitable TMMs.

Many TMMs have been proposed for ultrasound phantoms (Madsen *et al* 2005, Zell *et al* 2007, Troia *et al* 2017), but there is a need for new materials, and new methods to use them in phantoms. Currently-used TMMs include silicone (McLeod *et al* 2016), agar/gelatin (Pavan *et al* 2010), poly(vinyl alcohol) cryogel (Surry *et al* 2004, Xia *et al* 2011), polyvinyl chloride-plastisol (McLeod *et al* 2015), and urethane rubber (Culjat *et al* 2010). Identifying a TMM that allows for independent tuning of parameters such as the speed of sound, ultrasonic scattering, and attenuation, and which can be developed from non-toxic, mechanically robust, and inexpensive materials, has been particularly challenging. Longevity and low manufacturing costs are also important considerations. Silicone has been used for sophisticated patient-specific phantoms (Holmes *et al* 2008, McLeod *et al* 2012), but its attenuation is high (Browne *et al* 2003). Aqueous phantoms such as agar and gelatin gels can be tuned so that their ultrasonic properties are similar to those of tissue (Madsen *et al* 2005), but evaporation limits their longevity and these materials are not mechanically robust.

Recently, several studies have highlighted oil-based materials as promising TMMs. Oudry *et al* (2009) reported a mineral oil-based TMM that included styrene-ethylene/butylene-styrene (SEBS)—type copolymers to influence the phantoms’ elasticity. In a recent study by Cabrelli *et al* (2016), both the acoustic and optical properties of mineral-oil based mixtures were found to vary with the concentrations of SEBS and low-density polyethylene additives. Vieira *et al* (2013) used paraffin-gel wax TMMs based on mineral oil to develop heterogeneous phantoms for ultrasound-guided breast needle biopsies. Additives can provide acoustic backscattering; they include silica or graphite powder (Oudry *et al* 2009) and glass spheres (Vieira *et al* 2013, Cabrelli *et al* 2016). However, to the authors’ knowledge, there have not been any demonstrations of oil-based TMM phantoms with complex patient-specific geometries.

In this study, we demonstrate that the acoustic properties (acoustic attenuation and backscattering) of a mineral-oil based material called ‘gel wax’, can be tuned with paraffin wax and glass spheres additives. Additionally, we demonstrate, for the first time, ‘gel wax’ can be shaped with 3D printed moulds to create anatomically realistic phantoms that are derived from patient-specific anatomy. The developed phantoms cover a wide range of clinical fields including regional anesthesia, cardiac electrophysiology and fetal therapy.

## 2. Materials and methods

The methodology in this study had four components. First, we investigated how two additives to gel wax could be used to tune acoustic attenuation and backscattering. Second, we measured the Young’s modulus of native gel wax. Third, we designed three types of moulds for gel wax, and fabricated them with 3D printing. Finally, we fabricated the phantoms, and validated them with a clinical ultrasound imaging scanner.

### 2.1. Acoustic properties tuning and characterisation

In its native form, the gel wax used here (Product number: FF1 003, Mindsets Online, Waltham Cross, UK) attenuated and scattered ultrasound far less than typical biological tissues (see section 3). Glass spheres (Boud Minerals, Lincolnshire, UK) with a nominal diameter range of 0–63 *µ*m were added to increase acoustic backscattering, and paraffin wax (Alec Tiranti, London, UK) was added to increase acoustic attenuation.

#### 2.1.1. Gel wax material fabrication

To fabricate the sample mixtures, gel wax was first melted to a temperature greater than 30 °C beyond its melting point (*ca*. 65 °C), in a glass beaker with a heating plate. Glass spheres and/or paraffin wax were gradually added during continuous mechanical stirring until the solution was visually homogeneous. Subsequently, sonication (CL18, Fisher Scientific, Pittsburgh, PA; 120 W) was performed for 30 s (65% of maximum power) to avoid particle aggregation. After sonication, the solution was reheated and degassed for approximately 2 min until air bubbles were absent with visual inspection. It was then allowed to cool down, and then poured into different types of moulds, as described in the following sections.

#### 2.1.2. Acoustic properties characterisation

Two series of samples were fabricated with different concentrations of paraffin wax and glass spheres. In the first series, paraffin wax concentrations varied from 0 to 8 w/w% and no glass spheres were included. In the second series, the glass sphere concentration was varied from 0.05 to 0.9 w/w% and no paraffin wax was added. When melted, these samples were poured into rectangular moulds. These moulds were created with an acrylic spacer with a nominal thickness of either 2 mm or 5 mm, which was positioned between two glass microscope slides. This spacer had an opening that allowed for gel wax to be poured in between the two slides. The gel wax was cooled by placing it in room temperature water, with the spacer opening facing upward. For characterising acoustic properties, the glass slides were removed, so that a rectangular gel wax slab (*ca*. 35 mm  ×  50 mm) surrounded by the acrylic spacer remained. The speed of sound and acoustic attenuation were measured using an insertion method (Xia *et al* 2013) in reflection mode, with a single-piezo transducer (V312-SU, 10 MHz central frequency, Olympus, Shinjuku, Japan). The transducer and a metal reflector were mounted in deionized water at room temperature (*ca*. 20 °C), so that the acoustic axis of the transducer was perpendicular to the surface of the metal reflector. An ultrasonic pulser/receiver (5077PR, Olympus, Shinjuku, Japan) was used to drive the transducer and receive the echo signal from the reflector. The received echo signal was then digitized at 100 MS s^−1^ by a digital acquisition card (USB-5132, National Instruments, Austin, USA) and transferred to a personal computer for processing.

For each material composition, pulse-echo ultrasound signals (*S*_1_*, S*_2_) with a pair of two gel wax slabs with nominal thicknesses of 2 mm and 5 mm were acquired separately. Using tabulated values for the speed of sound and the frequency dependent acoustic attenuation of water, *c*_w_ and ${{\alpha }_{{\rm w}}}\left(\,f \right)$ (Bilaniuk and Wong 1993), the speed of sound (*c*_s_) was estimated by solving for this variable in equation (1):

1 \begin{align} \newcommand{\e}{{\rm e}} \displaystyle \Delta t=\frac{2({{d}_{2}}-{{d}_{1}})}{{{c}_{{\rm s}}}}-\frac{2({{d}_{2}}-{{d}_{1}})}{{{c}_{{\rm w}}}}\nonumber \end{align}

where *d*_1_ and *d*_2_ are the thicknesses of the sample pair (measurements performed on the surrounding rigid acrylic frames with digital callipers), and Δ*t* is the difference in the time-of-flights, which was calculated using cross-correlation of *S*_1_ and *S*_2_. Likewise, the acoustic attenuation of the sample, ${{\alpha }_{{\rm s}}}\left(\,f \right)$, was estimated using equation (2):

2 \begin{align} \newcommand{\e}{{\rm e}} \displaystyle {{\alpha }_{{\rm s}}}\left(\,f \right)=\frac{10}{{{d}_{2}}-{{d}_{1}}}{{\log }_{10}}\left[ \frac{{{I}_{1}}\left(\,f \right)}{{{I}_{2}}\left(\,f \right)} \right]+{{\alpha }_{{\rm w}}}\left(\,f \right)\nonumber \end{align}

where *I*_1_(*f*) and *I*_2_(*f*) are the power spectra of *S*_1_ and *S*_2_. With most materials, acoustic attenuation follows a power law of the form ${{\alpha }_{{\rm s}}}\left(\,f \right)={{\alpha }_{0}}{{f}^{n}}$, in which ${{\alpha }_{0}}$ is termed the pre-factor, and *n* a positive exponent. The estimated values of ${{\alpha }_{{\rm s}}}\left(\,f \right) ~ $ were fitted to the power law using a non-linear least squares regression algorithm (Matlab, Mathworks, Natick, NH) to estimate ${{\alpha }_{0}}$ and *n*. The water temperature (*T*), which was recorded using a digital thermometer (4378, Fisher Scientific, Pittsburgh, UK), was used to estimate *c*_w_ (Bilaniuk and Wong 1993). For each sample pair, 5 measurements of *S*_1_ and *S*_2_ were obtained from distinct regions. The acoustic impedance (*Z*) of native gel wax was calculated using measured densities of 10 samples. The density of each sample was obtained with a digital weight scale (BP211D; 0.01 mg nominal precision; Sartorius, Göttingen, Germany) and a digital calliper (CD-15APX;  ±20 *µ*m nominal precision; Mitutoyo, Kawasaki, Japan).

To assess the acoustic backscattering, we compared ultrasound images acquired from phantoms with varying concentration of glass spheres and 2 w/w% paraffin wax, and from the human arms of three healthy volunteers. The images were acquired using a clinical ultrasound scanner (SonixMDP, Analogic Ultrasound, Richmond, Canada) with a linear array imaging probe (bandwidth: 14–5 MHz; L14-5/38, Analogic Ultrasound, Richmond, BC, Canada). The ultrasound acquisition settings, which included excitation frequency, gain, depth, dynamic range and power, were consistent across images; the speed of sound used for reconstruction was different (gel wax phantoms: 1440 m s^−1^; human tissues: 1540 m s^−1^). For each image, the histogram, mean value and standard deviation of the image intensities were calculated. Prior to this, the acquired images were scaled globally so that the minimum and maximum across all images were 0 and 1, respectively.

### 2.2. Mechanical testing

Uniaxial compression test was performed to determine the Young’s modulus of native gel wax using a ZwickiLine testing machine (Zwick GmbH & Co, Ulm, Germany) equipped with a 250 N load cell. Melted gel wax material was casted in petri dishes (ca. 49 mm  ×  10 mm, Anumbra, FGH plus, Šumperk, Czech Republic) to fabricate 5 cylindrical samples. Each sample was placed between two acrylic plates (*ca*. 100 mm diameter) that were larger than the sample surface. The interface between the sample and the loading plates was lubricated with bran oil in order to minimise friction and to ensure homogeneous compression. A compression loading test with a pre-compression load of 0.25 N was performed at 10 mm min^−1^ rate at room temperature (*ca*. 25 °C) until the sample’s thickness was reduced by 60%. The applied forces were then converted into stress by assuming the sample’s volume remained constant during compression. The Young’s modulus was calculated by applying least squares linear fitting to the slope of the stress–strain curve from 0 to 40% deformation. Prior to each test, the dimensions of each sample were measured in 5 distinct locations using a digital caliper and the averaged value was used for the stress calculation.

### 2.3. Phantom creation and evaluation

#### 2.3.1. Nerve and vessel phantom

As a demonstration of the potential to create heterogeneous tissue types, a phantom comprising cylindrical nerve- and vessel-like structures was created. The gel wax material that represented nerves comprised 2 w/w% glass spheres and 5 w/w% paraffin, that which represented vessels had no additives, and that which represented surrounding tissue comprised 0.5 w/w% glass spheres and 2 w/w% paraffin wax. The choices for paraffin wax concentrations were made based on the acoustic attenuation contrasts between nerves (Chen *et al* 2014), blood vessels (Treeby *et al* 2011) and background tissue (Mast 2000); The nerves (5 mm in diameter) and vessels (5 mm in diameter) were first cast using a 3D printed cylindrical mould. When solidified and removed from the mould, they were hung vertically within the plastic mould (64  ×  48  ×  42 mm). Subsequently, the surrounding tissue gel wax material was first allowed to cool down to *ca*. 95 °C, and was then poured slowly around the vessel and nerve structures to minimize their deformations. Finally, the phantom was allowed to solidify at room temperature.

#### 2.3.2. Heart atrium phantom

An ultrasound phantom to represent tissue surrounding the cardiac atria and adjoining portions of the superior and inferior vena cavae was created with a homogeneous gel wax material. This material comprised 0.5 w/w% glass spheres without paraffin wax. The mould represented the blood-filled regions and included a handle to position it. It was 3D printed using the Ultimaker printer (Ultimaker 2 Plus, Ultimaker B.V., Geldermalsen, the Netherlands) with White Polymax Enhanced Poly Lactid Acid (PLA) material. The mould was coated with a thin layer of bran oil to facilitate removal of the cooled gel wax material. Positioned within a plastic container (135 mm  ×  135 mm  ×  75 mm), melted gel wax material was poured around the mould, up to the vertical midpoint. After the gel wax material was cooled, the mould was removed by pulling it vertically upwards, which left empty spaces that were subsequently filled with water as the blood-mimicking fluid. An empty cylindrical chamber 25 mm in diameter was created to allow for manual palpation of the left atrium, to simulate cardiac contractions.

The anatomy of the cardiac phantom was derived from an MRI scan of a healthy 30-year-old male volunteer (whole heart 3D balanced, steady-state free precession acquisition; 1.5 T Avanto MR scanner, Siemens Medical Solutions, Erlangen, Germany). Several digital post-processing steps were performed with different software packages to generate a mould from this image volume. They included isolation of the right and left atria (Netfabb, Autodesk, San Rafael, CA), smoothing of the surfaces of the data (Meshmixer, Autodesk, San Rafael, CA), creation of cylinders representing the venae cavae, addition of supporting structures (FreeCAD), and fusion of the different components of the mould (Blender, Blender foundation, Amsterdam, the Netherlands). It was ensured that the interatrial septum had a minimum thickness of 5 mm, so that this anatomical region was sufficiently robust to withstand removal of the mould. The mould was printed upside down, so that the surfaces designed for contact with the gel wax did not have printed support material connected to them.

#### 2.3.3. Placenta phantom

The chorionic surface of a human healthy term placenta was represented in an ultrasound phantom that included superficial fetal vessels. The placenta was collected with written informed consent after a caesarean section delivery at University College Hospital (UCH). The Joint UCL/UCLH Committees on the Ethics of Human Research approved the study (14/LO/0863). As the first fabrication step, large vessels were digitally traced in two dimensions from a photograph using vector graphics software (Inkscape 2016, (Online)), and 3D drawing software (FreeCAD 2016 (Online)) was used to extend it into a 3D vessel model with separate arterial and venous components. The diameters of the arteries ranged from 5 mm at the umbilical cord insertion to 2 mm at the smallest branches in the vessel model. The diameters of the veins were double those of the arteries (Clark *et al* 2015). The vessel model was incorporated into two moulds that were 3D printed (Formlabs, Somerville, USA). The arterial mould (lateral dimensions: 92 mm  ×  99 mm) and the venous mould (105  ×  97 mm) each comprised two parts, which bisected the spaces for vessels; these parts were aligned and held in place with screws.

Vessels were created by melting pure gel wax at 200 °C in a glass beaker, placing it in a vacuum chamber (Thermo Scientific, Nalgene) to remove the air bubbles, and then injecting it through holes in the sides of the moulds with a 30 ml clinical syringe. To allow for arteries to be visually differentiated from veins, red and blue liquid candle dyes (The Candle Making Shop, UK) were added to the gel wax. Veins and arteries were created from separate batches of gel wax equal in weight. For arteries, 10 drops (*ca*. 4 ml) of blue dye were added to 143 g gel wax; for veins, 10 drops of red dye and two drops of arctic blue candle dye were added.

To create the underlying placental base, glass spheres (0.5 w/w%) and 6 drops of red dye were added to 400 g of pure gel wax and the mixture was heated to 220 °C. The mixture was placed in the vacuum chamber to remove the air bubbles, poured into a square container (94 mm  ×  100 mm), and then left to partially cool. The veins were placed on the top of the base, and the arteries were placed above them. With the base only partially cooled, the vessels sunk into the placenta, thereby forming a single phantom.

#### 2.3.4. Ultrasound imaging

Ultrasound imaging of the phantoms was performed with a clinical scanner (MDP, Ultrasonix, Richmond, Canada) using a linear array imaging probe with a nominal bandwidth of 5–14 MHz (L14-5). For the nerve and vessel phantom, ultrasonic gel was used as a coupling medium; for the cardiac and placenta phantoms, water was used. For all imaging, the value of the speed of sound used for reconstruction was 1430 m s^−1^.

## 3. Results

**Figure 1.**
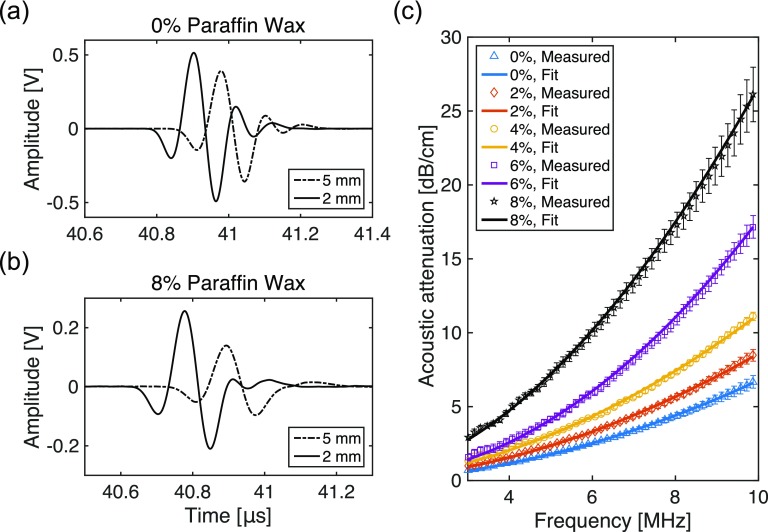
Acoustic attenuation of gel wax samples. Pulse-echo ultrasound signals acquired with two pairs of gel wax slabs (5 mm and 2 mm), with paraffin wax concentrations of 0% and 8%, are shown in (a) and (b), respectively. The measured acoustic attenuation of gel wax samples increased with paraffin wax concentration, and followed the frequency power law (${{\alpha }_{0}}{{f}^{n}}$) over a range of 3–10 MHz (c). For each paraffin wax concentration, the measured data represent average values from five repeated measurements; the error bars represent the standard deviations. The fit curve for each concentration is derived from the mean values for ${{\alpha }_{0}}$ and *n* that resulted from five repeated measurements. The values of ${{\alpha }_{0}}$ and *n* are listed in table 1.

The addition of paraffin wax had a strong effect on acoustic attenuation and a weak effect on the speed of sound and acoustic impedance. The acoustic attenuation increased rapidly with the paraffin wax concentration (figure 1; table 1). The frequency dependence of the attenuation was found to be well modelled by the power law. As the concentration of added paraffin wax was increased to 8% of the native weight, the attenuation increased by 308% at 3 MHz from 0.72 to 2.91 dB cm^−1^ and by 289% at 10 MHz from 6.84 to 26.63 dB cm^−1^. These ranges of attenuation contain reported values of most soft tissues (Mast 2000, Culjat *et al* 2010). Over the same concentration range, the speed of sound was not substantially affected (table 1). At 1448.5 m s^−1^, the speed of sound of native gel wax was 6.3% lower than average speed of sound for soft tissue (1540 m s^−1^), which is a typical value used for reconstruction in most clinical ultrasound imaging scanners. The measured acoustic impedance of native gel wax, 1.22  ±  0.01 kg m^−2^ s^−1^ $\times $ 10^6^, was lower than that of typical soft tissue (see 1.62 kg m^−2^ s^−1^ $\times $ 10^6^ for muscle (Mast 2000)).

**Table 1.** Measured acoustic properties of gel wax, in which the only additive was paraffin wax. Each property (mean  ±  standard deviation) was obtained from measurements from five distinct regions. Acoustic attenuation as a function of frequency (*f*) is depicted by the power law curve (${{\alpha }_{0}}{{f}^{n}}$), with parameters obtained from non-linear least squares fitting.

Paraffin wax concentration (w/w%)	Acoustic attenuation ${{\alpha }_{0}}{{f}^{n}}$ (dB cm^−1^)		Speed of sound *c*_s_ (m s^−1^)
	${{\alpha }_{0}}$	$~n$	
0	0.080 ± 0.007	1.93 ± 0.06	1448.5 ± 0.8
2	0.117 ± 0.004	1.86 ± 0.03	1447.9 ± 0.5
4	0.156 ± 0.003	1.86 ± 0.01	1447.9 ± 1.1
6	0.142 ± 0.022	2.09 ± 0.07	1448.4 ± 1.7
8	0.348 ± 0.063	1.88 ± 0.10	1443.1 ± 1.5

**Figure 2.**
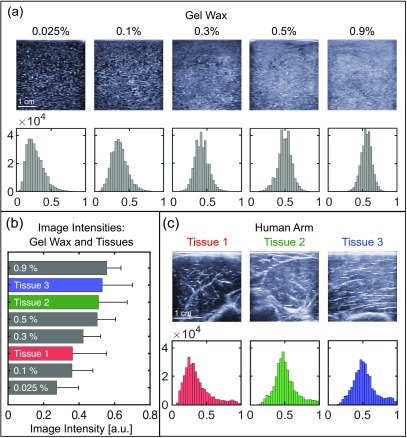
Acoustic backscattering of gel wax samples in comparison with tissues. (a) Ultrasound images and histograms of gel wax samples comprising 2 w/w% paraffin wax and different glass sphere concentrations. (b) Comparison of mean image intensity values of ultrasound images from gel wax samples and tissues. The error bars represent standard deviations. (c) Ultrasound images and histograms of human arm tissues. The ultrasound acquisition settings in (a) and (c) were consistent; only the speed of sound used for reconstruction was different (gel wax: 1440 m s^−1^, human tissues: 1540 m s^−1^).

The addition of glass spheres to provide acoustic backscattering had a prominent effect on the ultrasonic appearance, and it did not substantially affect the speed of sound and acoustic attenuation (table 2). The ultrasound images from gel wax phantoms and human arm tissues had similar image intensity distributions (figures 2(a) and (c)). The mean value of image intensities for gel wax phantoms increased with glass spheres concentration from 0.27  ±  0.12 at 0.025 w/w% to 0.56  ±  0.08 at 0.9 w/w% (figure 2(b)). The mean values of human arm tissues ranged from 0.36  ±  0.19 to 0.53  ±  0.17. At a concentration of 0.025 w/w%, the mean value was lower than the imaged human arm tissues, and at 0.9 w/w% it was higher. The standard deviations of the image intensity values for gel wax phantoms were slightly lower that human arm tissues; this difference can be attributed to the heterogeneity of human arm tissues. Our assessment suggested that 0.5 w/w% provides an acoustic backscattering appearance similar to that of soft tissues.

**Table 2.** Measured acoustic properties of gel wax, in which the only additive was glass spheres. Each property (mean  ±  standard deviation) was obtained from measurements from five distinct regions. Acoustic attenuation as a function of frequency (*f*) was well described by the power law (${{\alpha }_{0}}{{f}^{n}}$), with parameters obtained from non-linear least squares fitting.

Glass spheres concentration (w/w%)	Acoustic attenuation ${{\alpha }_{0}}{{f}^{n}}$ (dB cm^−1^)		Speed of sound *c*_s_ (m s^−1^)
	${{\alpha }_{0}}$	$~n$	
0	0.111 ± 0.013	1.80 ± 0.07	1445.7 ± 0.7
0.05	0.108 ± 0.012	1.83 ± 0.05	1443.9 ± 0.6
0.1	0.102 ± 0.005	1.87 ± 0.06	1443.5 ± 0.7
0.5	0.106 ± 0.028	1.88 ± 0.11	1443.4 ± 0.3
0.9	0.113 ± 0.009	1.87 ± 0.05	1444.1 ± 0.8

Native gel wax was sufficiently robust to maintain its integrity in the presence of compressive deformations up to 60%. Its stress–strain curve was linear (*R*^2^  >  0.99) for deformations up to 40%, with a Young’s modulus of 17.4  ±  1.4 kPa. For greater deformations, the stress–strain curve was non-linear, with the derivate tripling from 40% to 60%.

**Figure 3.**
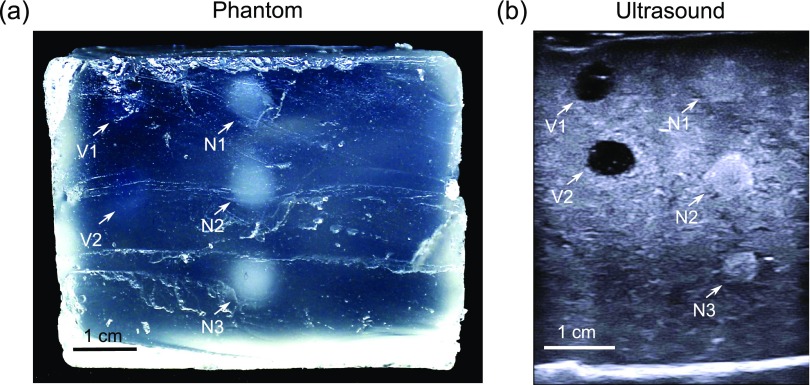
Nerve and vessel phantom. (a) Photograph showing the cross-sectional view obtained after cutting through the phantom. The nerves (N1, N2, N3) are translucent, and can be visually identified from a transparent background; the vessels (V1, V2) are barely visible. (b) An ultrasound image of the phantom, in which the blood vessels present as hypoechoic and the nerves present as hyperechoic.

In the nerve and blood vessel phantom, all embedded structures were visible on ultrasound (figure 3). The blood vessels presented as hypoechoic; the nerves, as hyperechoic with a slight difference relative to the background that was reminiscent of that seen in some clinical contexts. Slight deviations from circularity were apparent in both vessels and nerves, which may have arisen from heating of these structures during embedding within the surrounding melted background gel wax during fabrication. The diameters of the vessel and nerves, as measured on the ultrasound images, ranged from 4.3 to 6.3 mm. The mean value of 5.6 mm was 12% different from that of the moulds from which they originated. The background had a largely homogeneous appearance but some scatters were present, which may have corresponded to small air bubbles. There was an absence of bubbles around the vessels and nerves. Moreover, no artefacts or prominent acoustic changes at interfaces with the background were apparent.

**Figure 4.**
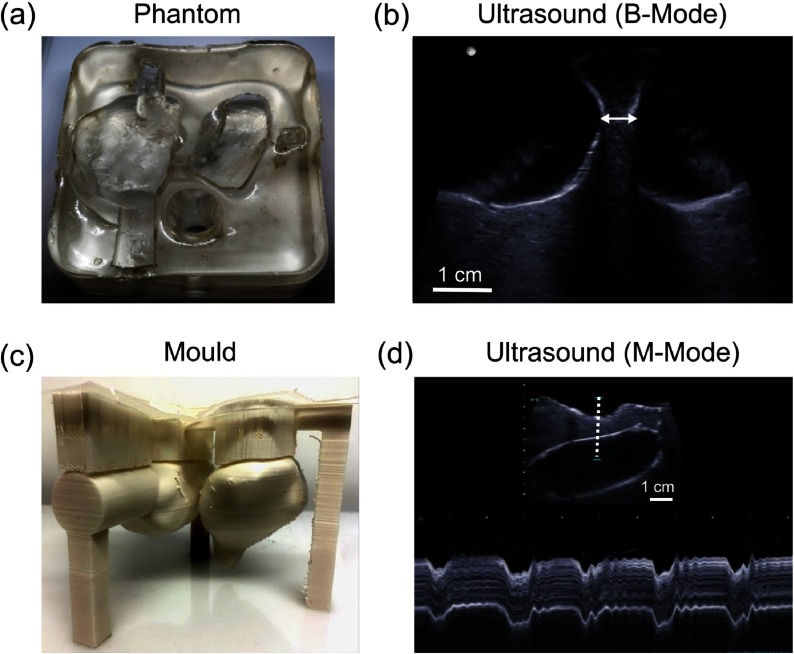
Cardiac phantom. (a) Photograph (top view) without water in the heart chambers. (b) B-mode ultrasound (US) image of the septum, with the heart chambers filled with water. The septum was intact, with a minimum measured thickness of 6.6 mm (arrow). (c) The 3D printed mould used to create the phantom. (d) M-mode US image of the septum, acquired from a region indicated by the dashed line in the inset US B-mode image, which shows periodic deformation of the septum induced by manual palpation.

In the cardiac phantom, the surfaces of the gel wax that formed the heart chambers retained the shapes of the corresponding 3D printed mould (figure 4). In particular, the septum was intact, despite having a minimum measured thickness of only 6.6 mm. The interface between the gel wax and the water that filled the heart chambers was slightly more hyperechoic than the tissue-blood interface encountered in clinical practice. The underlying gel wax had a largely homogeneous appearance that resembled the human myocardium. Due to the compliant nature of gel wax, the phantom was readily deformed with palpation, and these deformations were visualised with M-mode imaging.

**Figure 5.**
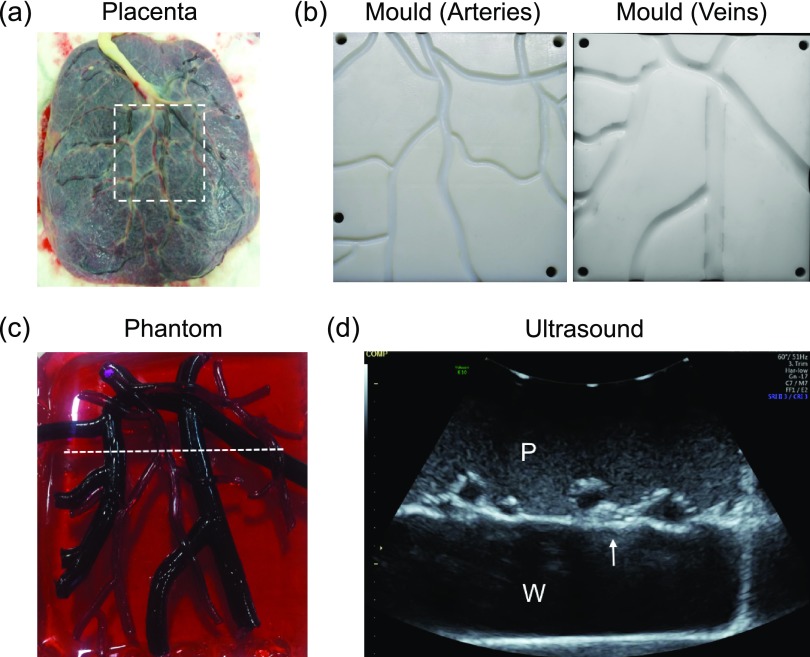
Placenta phantom. (a) Photograph of the chorionic surface of a human placenta on which the phantom was based. (b) 3D printed moulds for creation of chorionic arterial and venous vasculature, derived from the human placenta (dashed region in (a); 92 mm  ×  99 mm). (c) Photograph of the placenta phantom. The vasculature created from the moulds shown in (b) was stretched over the rectangular placental base (94 mm  ×  100 mm). (d) Ultrasound image of the placenta, corresponding to the dashed white line in (c). The placenta was imaged in water, with the ultrasound probe in contact with the maternal side of the placenta, as is the case in transabdominal ultrasound imaging of the pregnant uterus. The chorionic superficial fetal vessels (arrow) were clearly apparent with hypoechoic centres and hyperechoic boundaries that were recessed from the chorionic surface of the placenta. P: placenta; W: water.

In the placenta phantom, the visual and ultrasonic appearances of the chorionic vessels and underlying chorionic tissue strongly resembled those of the human placenta from which they were derived, according to A.L.D., consultant specialist in fetal medicine. With ultrasound imaging, the superficial chorionic fetal vessels were clearly apparent with hypoechoic centres and hyperechoic boundaries that were recessed from the chorionic surface of the placenta (figure 5). The hyperechoic boundaries of these surface vessels and the chorionic surface of the placenta were larger than those encountered in clinical practice. The background was speckled and largely homogeneous. Visually, the veins were much darker than the arteries; the background was translucent. The gel wax proved to be sufficiently compliant to allow for the arteries and veins to cross over each other, a situation that frequently occurs in the human placenta.

## 4. Discussion

Here, we demonstrated for the first time that heterogeneous phantoms with anatomical realism can be generated from gel wax. We found that gel wax is well suited to moulding with 3D printed plastics due to its favourable mechanical properties. These moulds can readily be derived directly from tissue to create patient-specific phantoms. Acoustic attenuation, speed of sound and acoustic backscattering were obtained within the range of values quoted for soft tissues by varying concentrations of two additives: paraffin wax and glass spheres.

The speed of sound and the acoustic impedance of gel wax are lower than that of most soft tissues and water (Mast 2000, Culjat *et al* 2010). One consequence is that immersion of gel wax phantoms in water can lead to hyperechoic boundaries on ultrasound imaging. These challenges could be mitigated by the use of different surrounding fluids, such as mineral oil. Furthermore, the low speed of sound could affect the scaling of the images during reconstruction. Copolymers could be added to modify the speed of sound of the material (Oudry *et al* 2009). Here, frequency dependent acoustic attenuation involved a power law exponent (*n*  ≈  1.9) that falls within the range of values reported for different soft tissue types (1.0–7.4) (Duck 1990, Azhari 2010 (appendix A)). As a result, tuning the acoustic attenuation of gel wax to match specific soft tissue types within a broad frequency range may be challenging.

The native gel wax used in this study is readily deformable. Indeed, its Young’s modulus is similar to the average values of 18–22 kPa reported for normal breast fat tissue (Krouskop *et al* 1998). Its stiffness could be increased significantly by incorporating additives such as paraffin wax and copolymers (Oudry *et al* 2009). These additives could reduce the range of deformations over which the stress–strain relationship is substantially linear, which could be of interest to introduce non-linearities encountered in soft-tissues during ultrasound elastography studies (Pavan *et al* 2010).

There are several ways in which the anatomically realistic ultrasound gel wax phantoms in this study could be improved. First, spatial variations in glass sphere concentrations were visible within regions that were designed to be homogenous. These variations likely arose from differences in the densities of glass spheres and the surrounding gel wax. Increasing the mixing speed and increasing the rate of cooling of the gel wax after it is poured in the mould will likely reduce heterogeneities but it might also introduce air bubbles. To mitigate the risk of air bubble formation, it may be advantageous to mix and pour gel wax within low pressure chambers. Second, the acoustic properties (acoustic attenuation and back scattering) in this study were assigned to provide contrast between the different tissue structures, such as blood vessels and underlying background. In future work, acoustic properties values could be assigned to represent specific tissue types.

In this study, anatomically realistic ultrasound phantoms with feature sizes as small as 2 mm were created using gel wax with 3D printed moulds. Direct printing of gel wax could be useful to create structures with greater 3D complexity. One recent step in this direction was provided by Dong *et al* (2015), who developed a fuse deposition modelling system to fabricate 3D heterogeneous phantoms for optical imaging. Gel wax was mixed with additives and directly printed using a custom print head with multiple channels and an embedded mixing device. We conclude that gel wax is a material which could lead to a new generation of ultrasound phantoms with a step change in anatomical realism.
